# The Scandinavian Displaced Lateral Clavicle trial (ScanDiLaC): a study protocol for a randomized clinical trial

**DOI:** 10.1186/s13063-026-09844-8

**Published:** 2026-06-13

**Authors:** Pontus Christersson, Dennis Karimi, Kirsten Grønhaug, Antti Launonen, Stig Brorson, Frede Frihagen, Bjarke Viberg, Nils Hailer, Olof Wolf

**Affiliations:** 1 Uppsala University Hospital, Uppsala, Sweden; 2 Trauma Orthopaedic Research Copenhagen Hvidovre (TORCH), Department of Orthopaedic Surgery, Copenhagen University Hospital Hvidovre, Copenhagen, Denmark; 3 Sykehuset Østfold, Grålum, Norway; 4 Tampere University Hospital, Tampere, Finland; 5 Zealand University Hospital, Køge, Denmark; 6 Odense University Hospital, Odense, Denmark

**Keywords:** Lateral clavicle fracture, Distal clavicle fracture, Nonsurgical, Surgical fixation, Randomized controlled trial, Patient-reported outcomes

## Abstract

**Background:**

Evidence regarding the treatment of displaced, extraarticular lateral clavicle fractures is scarce. No study has shown clinically significant differences between surgical and nonsurgical treatment, but the sample sizes have been small, as it has been difficult to include enough patients with this relatively uncommon fracture type.

**Purpose:**

This study aims to compare outcomes after surgical and nonsurgical treatment for displaced lateral clavicle fractures.

**Methods:**

This is a pragmatic, noninferiority, preference-tolerant, randomized controlled trial (RCT). A total of 100 patients between the ages of 18 and 65 with displaced lateral clavicle fractures will be randomly allocated on a 1:1 ratio to surgical or nonsurgical treatment with the option of early crossover after 6 weeks. An observational cohort will comprise patients not willing to be randomized. This is a multicenter Scandinavian RCT including hospitals in Sweden, Norway, Denmark, and Finland. The primary outcome is the Disabilities of the Arm, Shoulder and Hand (DASH) score at 1 year. Follow-up points will be 6 weeks, 3 months, 6 months, and 1 year. The secondary outcomes are the DASH score, the EQ-5D-5L score, the University of California, Los Angeles (UCLA) activity score, the Nottingham Clavicle Score (NCS), a visual analog scale (VAS) for pain, and anchor questions in the form of the Patient Global Impression of Change (PGIC) collected at all timepoints during the study. All complications, radiographic healing, and return to work will be reported.

**Discussion:**

The optimal treatment for displaced Neer type II and V lateral clavicle fractures remains a topic of debate. This RCT may provide a better understanding of the differences in outcomes of nonsurgical and surgical treatment while reflecting real-world clinical practice and guiding the development of a treatment algorithm for the orthopedic community.

**Trial registration:**

ClinicalTrials.gov NCT06981065. Registered on 19 May 2025. https://clinicaltrials.gov/study/NCT06981065?term=Scandilac&rank=1

**Supplementary Information:**

The online version contains supplementary material available at 10.1186/s13063-026-09844-8.

## Background and rationale

Clavicle fractures constitute 3–4% of all fractures in adults [1, 2]. Midshaft fractures are most common (65–82%) followed by lateral (12–30%) and finally medial (2–6%) fractures. Most lateral clavicle fractures (LCFs) are extraarticular. There is a typical bimodal pattern consisting of young males and elderly women. The injury pattern is often sports-related (dominated by cycling) or traffic accidents in the young or simple falls in the elderly [3–6]. LCFs are often classified according to Neer, modified by Craig, where types II and V are unstable and equivalent to a coracoclavicular (CC) disruption (see Supplementary material 1, S1) [7, 8].

High-quality trials comparing treatment of displaced extraarticular lateral clavicle fractures (LCFs) are scarce. No published RCT has included enough patients to provide sufficient statistical power to detect clinically meaningful differences.

The desire to restore the anatomy and to avoid nonunion have been the main reasons for surgical treatment of displaced LCFs. Nonunion rate after nonsurgical treatment has been reported as high as 33% [7–10]. Surgical treatment of an LCF commonly consists of plate fixation (anatomical locking plate or hook plate) with or without CC fixation [11–17]. Complications after surgical treatment are relatively common, particularly when using hook plates, which can cause subacromial erosion, osteolysis, and rotator cuff injury [18–21]. Hook plate removal is advocated after about 3 months, and many regard this secondary procedure as part of the treatment algorithm [22–24].

However, several systematic reviews and meta-analyses have reported no difference in patient-reported outcome measures (PROMs) between surgical and nonsurgical treatment, even in cases with radiological nonunion [10, 25, 26]. Two RCTs have attempted to provide evidence in this regard, but both were underpowered [27].

The aim of this study is to determine if nonsurgical treatment is noninferior to surgical treatment of Neer type II and V displaced LCFs.

## Methods: participants, interventions, comparisons, and outcomes

### Objectives

To compare PROMs of patients allocated to surgical or nonsurgical treatment of displaced Neer type II and V LCFs, including early identification and treatment of potential symptomatic delayed union.

### Trial design

A pragmatic, multicenter, randomized, outcome assessor-blinded, noninferiority trial. The ScanDiLaC protocol conforms with the Standard Protocol Items: Recommendations for Interventional Trials (SPIRIT) [28]. Patients not randomized and treated according to preference will be offered inclusion in an observational cohort.

### Study setting

Sites from Sweden, Denmark, Norway, and Finland were recruited. They span from level I to III trauma centers. The Trial Steering Committee (TSC) consists of representatives from all participating countries.

The initial recruiting hospitals are summarized in Supplementary material 2 (S2) (with the expectation that the list will grow as interest and awareness of the study increases).

### Participants

The trial population will consist of adults aged 18–65 years presenting to one of the participating centers with Neer type II or V displaced LCF, without contraindications to either of the treatment arms (Table 1). Participants must provide informed consent and be able to complete follow-up including questionnaires in the Scandinavian languages or English.

**Table 1:** Inclusion and exclusion criteria of the ScanDiLaC trial

Inclusion criteria
Displaced Neer type II or V LCFs—fractures extending from the midshaft of the clavicle can be included if the majority of the fracture is located in the lateral part
Patient aged 18–65 years
Exclusion criteria
Same-time injury in the upper extremity estimated to affect the primary outcome
Polytrauma*
Pathological fracture
Open fracture
Neurovascular injury
Not treatable within 21 days of injury
Contraindications to anesthesia or surgery due to comorbidities
Unable to give informed consent (such as dementia, unwillingness to participate, mental disabilities)
Inability to complete follow-up, such as not being able to fill out questionnaires in Swedish, Danish, Norwegian, Finnish, or English)

^*^As defined by the New Berlin definition [29], see Supplementary material 3 (S3)

The lateral part of the clavicle extends from the medial border of the coracoid process to the acromioclavicular joint. Displacement varies greatly with body position, muscle tension, and radiographic view. Because of this, a pragmatic approach will be taken, meaning that fractures with any displacement can be included, and it will be up to each center to determine if a fracture is displaced and eligible for inclusion. Radiographs will be uploaded in the data collection software to allow central review for validity of inclusion.

#### Recruitment

Patients admitted to the emergency department at any of the trial sites will be clinically examined, and radiographs will be obtained to confirm the diagnosis. If the inclusion criteria are met and no obvious exclusion criterion is present, the patient will be given the study information which contains all relevant information pertaining to the study, a simple sling to rest the injured arm, and then be scheduled for an outpatient appointment with a physician active in the study recruitment within 10 days. At this visit, the patient is informed about the study and given time to ask questions about study procedures. Upon documented informed consent in the data collection software, randomization is performed.

#### Randomization

**Fig. 1:**
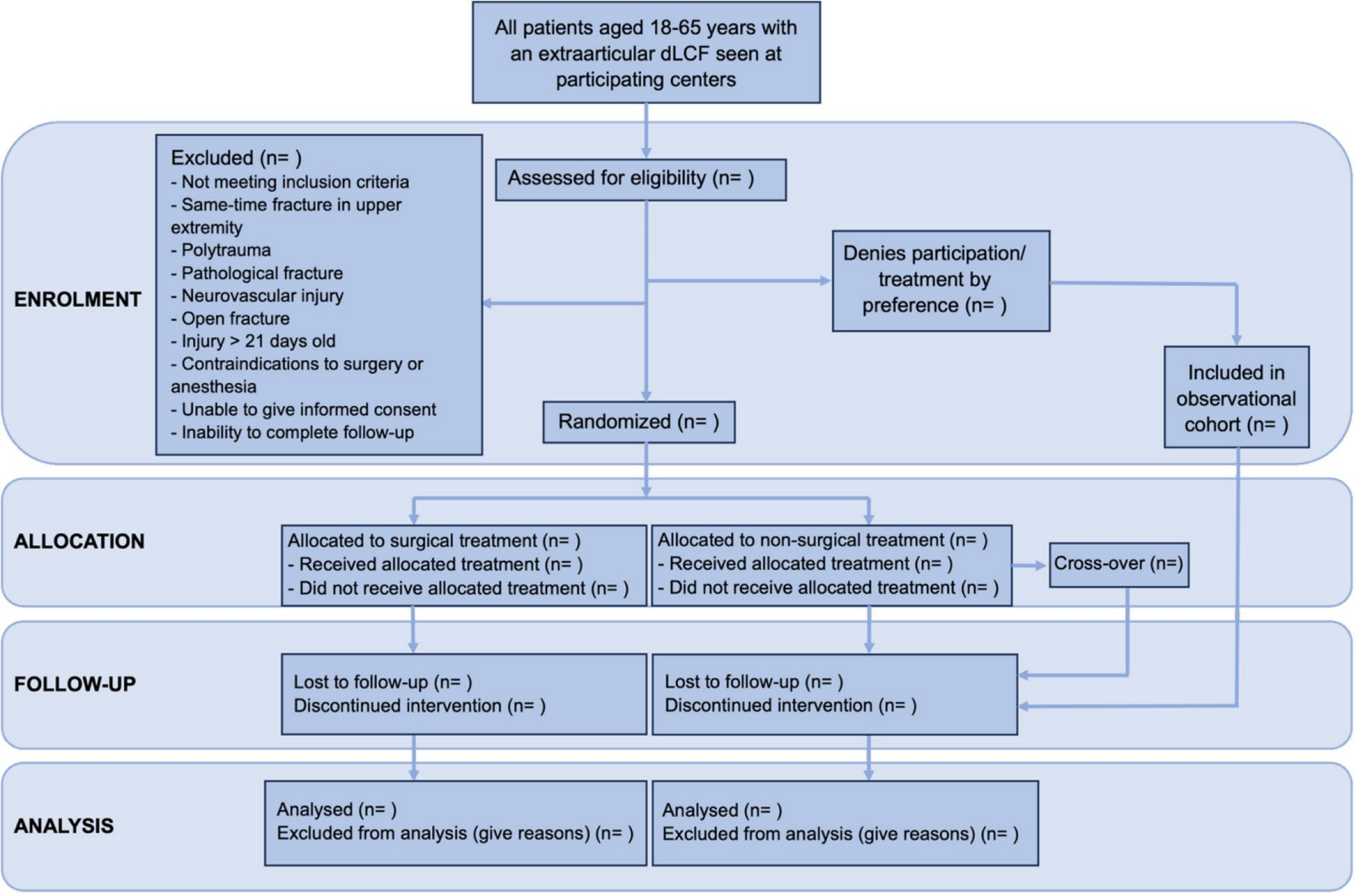
Trial flowchart for the ScanDiLaC study. dLCF, displaced lateral clavicle fracture

Research Electronic Data Capture (REDCap©) is a computerized database software hosted by Uppsala University [30]. It will be used to perform block randomization through an irreversibly random allocation sequence with selected block sizes of 2 and 4, stratified by site. Patients will be allocated in a 1:1 ratio between surgical and nonsurgical treatment. The trial worker who includes the patient will then acquire the allocated treatment from REDCap and initiate the treatment, either by scheduling the patient for surgery or commencing nonsurgical treatment according to the study protocol. The trial flowchart for the ScanDiLaC study is seen below (Fig. 1).

### Interventions and comparison

The interventions below are reported according to the TIDieR checklist [31]. The common recommended physical therapy protocol for both groups (see Supplementary material 4 and 5, S4, S5) is based on the Swedish physiotherapy program Axelina®, which is a standardized protocol for treating various conditions in the shoulder girdle [32].

#### Nonsurgical treatment

Appropriate nonsurgical treatment will be performed according to a standardized common physiotherapy protocol recommended for both interventions, with an initial period of immobilization consisting of a simple sling followed by structured physiotherapy. The sling is worn until pain resolves and when careful range of motion exercises can commence under the supervision of a licensed physiotherapist. The physiotherapy consists of home exercises combined with face-to-face visits with the physiotherapist if needed. The frequency and timing will be a shared decision between the patient and the physiotherapist. Adherence to physical therapy will be controlled by the physician during follow-up visits and recorded in REDCap.

#### Surgical treatment

The method of fixation can include plates (anatomic locking plate or hook plate) and screws ± CC fixation or CC fixation alone [33]. The plate will be applied via a supraclavicular approach to the lateral clavicle with screws in the proximal and distal fragments following basic principles of fracture fixation. The exact technique, type of plate, and CC fixation will be the treating surgeon’s choice and will be recorded.

Postoperatively, the arm will be placed in a sling, and unloaded exercises will commence from day one. The sling is initially used for comfort and can often be discarded approximately 1 to 2 weeks postoperatively. The type of sling and continued postoperative exercise regimen and physiotherapy will continue according to the recommended rehabilitation protocol, as described in the nonsurgical treatment above.

The level of experience of the operating surgeon will not be defined, but they must be familiar and experienced with the fixation method and material they are using. Each participating center will take responsibility to ensure that this is followed.

#### Crossover

In line with recent evidence regarding midshaft clavicle fractures and the development of symptomatic nonunion [34, 35], we intend to include the option of crossover to surgical treatment at the 6-week follow-up visit in the nonsurgical study arm if there are signs of the patient being at risk for symptomatic nonunion (severe persisting pain, no healing on radiographs) and if the patient requests surgery. Time from decision to surgery should be within 3 weeks.

### Outcomes

#### Data collection

All data apart from PROMs (which are automatically sent out to participants via e-mail) is entered directly into REDCap© by a trial worker directly involved in the trial.

#### Outcome timepoints

**Fig. 2:**
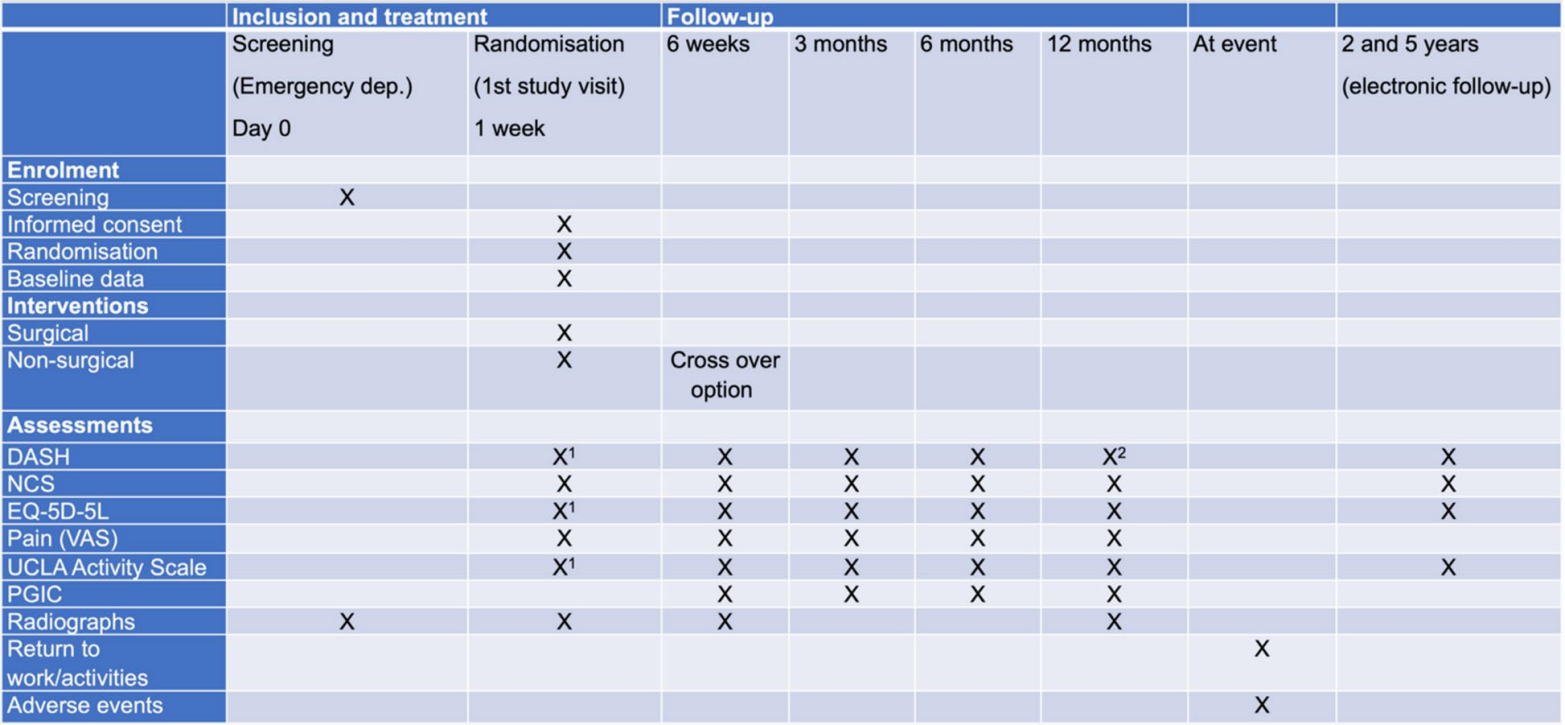
Study follow-up visits. DASH, Disabilities of the Arm, Shoulder and Hand; NCS, Nottingham Clavicle Score; EQ-5D-5L, European Quality of Life—5 Dimensions, 5 Levels; VAS, visual analog scale; UCLA, University of California, Los Angeles; PGIC, Patient Global Impression of Change; X1, preinjury scores; X2, primary outcome

Outcome measures will be obtained at the following timepoints: preinjury, baseline, 6 weeks, 3 months, 6 months, 1 year, 2 years, and 5 years. Study follow-up visits for the ScanDiLaC study is seen below (Fig. 2).

#### Baseline data

The baseline data collected includes patient demographics such as sex, age, work (and work status), arm dominance, height/weight, tobacco and alcohol habits, and list of current diseases, American Society of Anesthesiologists (ASA) grade, mechanism of injury, radiographs, previous surgeries and injuries in the affected shoulder. The following baseline questionnaires will be filled in digitally: Preinjury Disabilities of the Arm, Shoulder and Hand (DASH), University of California, Los Angeles (UCLA) activity scale, European Quality of Life, 5 Dimensions, 5 Levels (EQ-5D-5L) scores. Shoulder pain via an 11-point visual analog scale (VAS).

#### Primary outcome measure

The DASH score at 1 year.

For the upper extremity, the DASH score is the most used general PROM and it is validated and reliable for use in a multitude of pathologies in the upper extremity [36–38]. It has been validated for use in several upper extremity disorders [39] and has been cross-culturally adapted into Swedish, Danish, Norwegian, and Finnish populations [40].

#### Secondary outcome measures

- Upper extremity function and symptoms will be measured by the DASH at all timepoints.
- NCS at all timepoints.
- General health status questionnaire measured by the EQ-5D-5L.
- Shoulder pain will be assessed by an 11-point VAS, where patients are asked to assess their overall pain in the arm from 0 to 10 in a day.
- Healing will be assessed via routine radiographs by the participating centers at 1 year. Radiographic union is defined as cortical bridging between the medial and lateral fragments. Symptomatic nonunion is defined as a lack of radiographic union in combination with clinical signs of nonunion (movement at the fracture site, persisting pain) [41]. Radiographic nonunion without symptoms will be recorded but not considered an adverse event.
- Malunion of some degree is expected with nonsurgically treated clavicles and is therefore characterized by being symptomatic or not [42].
- Return to work collected from participants at all timepoints.
- Anchor questions in the form of the PGIC at all timepoints presented with 7 response options and analyzed as a 7-point Likert scale.
- Adverse events (AEs) after treatment will be recorded and include (see Table 2).

**Table 2:** Safety outcomes

Adverse event		
Surgical		Non-surgical
Deep infection (FRI)	Vascular injury or injury to brachial plexus	Symptomatic nonunion**
Superficial infection	Fixation failure	ACJ arthritis
Hardware irritation	Nonunion	Impingement
Supraclavicular nerve injury*	Scar or wound issues	
Serious adverse events (SAEs)—defined by the US Food and Drug Administration (FDA) [43] as any untoward medical occurrence that results in death, is life-threatening, results in hospitalization, significant disability, or permanent damage, or requires intervention to prevent impairment or damage. Major adverse cardiovascular events (MACEs) is a type of SAE that is defined as acute myocardial failure (AMI), stroke, cardiovascular death, heart failure (HF), and unstable angina		

^*^Prevalence varies greatly between studies [44, 45]

^**^Asymptomatic nonunion is not counted as an adverse event for the purpose of this study but will be recorded

For the purpose of this study, we will grade the severity of complications into five levels, according to the modified Clavien–Dindo classification [46], which was originally aimed at gastroabdominal surgical complications, but it has been adapted for use in orthopedic complications with a simplified severity grading I–V, where V is death [47]. They do not include complications pertaining to anesthesia.

#### Exploratory outcomes

Health economics through cost-effectiveness will be determined via the EQ-5D-5L, which performs a cost-utility analysis (CUA) to assess the improvement of each treatment in quality-adjusted life years (QALYs), as recommended by the National Institute for Health and Clinical Excellence (NICE) [48]. The level of activity according to the UCLA activity score will be recorded.

The observational cohort for patients who decline randomization. These patients will receive standard treatment at their local department and receive questionnaires at the same timepoints as the randomized cohort.

## Patient and public involvement

In the absence of a fracture patient organization, we have had contact with the Patient Organization for Osteoporosis, South Sweden, who have been given opportunity to have opinions on the study protocol.

We conducted semistructured qualitative interviews with patients recently treated for a clavicle fracture at Uppsala University Hospital, to assess their experiences about the treatment and hear their thoughts on the gross outline of the ScanDiLaC study. In line with previous qualitative studies on fractures [49], more information was generally requested regarding both the fracture itself and the treatment options. All patients were positive about the ScanDiLaC study’s design and using a data collection platform like REDCap. Some suggested that questionnaires be available in physical form to maximize participation in the older population, as well as in a phone application or text messaging format, both of which are possible with REDCap.

With these qualitative data and support from a patient organization, we judge the study design to be well optimized for patient participation and to minimize drop-out. We will aim to have physical copies available for all questionnaires at follow-up visits at all recruitment sites.

## Statistical methods

### Hypothesis

Null hypothesis (H0): The DASH score at 1 year after nonsurgical treatment is inferior to surgical treatment with a plate ± CC fixation of displaced, extraarticular LCFs.

Alternative hypothesis (HA): The DASH score at 1 year after nonsurgical treatment is not inferior to surgical treatment with a plate ± CC fixation of displaced, extraarticular LCFs.

### Sample size

To make an accurate sample size prediction, estimates in standard deviation (SD) scores for the DASH were acquired for the study population. As we found no study that explicitly studied these values for the DASH in patients with LCFs, a number of sources were used, including several RCTs [27, 50–52], as well as studies on different populations of the USA [53], Norway [54], and Germany [55]. Using the numbers from the studies above, we estimated that the mean SD for DASH is 14.3 for those aged 18–65 years. The minimal clinically important difference (MCID) was estimated using an extensive literature review and estimated as 10.4 [37–39, 56–61].

A sample size calculation was performed by a biostatistician at Uppsala Clinical Research Center (UCR) for the study, assuming a 20% attrition rate (an estimate on the basis of data from the SWIFFT trial (where a similar multicenter, pragmatic design was used) [62], the FISH trial [50], and the SHAFT trial [51]).

Since we have limited data on the correlations between the baseline and on-treatment DASH values, we calculate power for an unadjusted t-test, which approximates the primary baseline-adjusted mixed-effect model under a pessimistic scenario and provides a conservative estimate. Assuming a noninferiority limit of 10.4 (MCID), a one-sided alpha of 0.025, 90% power, and SD values of 14.3, a study population of 80 is needed. With an expected attrition of 20%, the total population becomes 100. The calculations were performed via R v. 4.2.3 and the base stats package.

### Statistical analysis

The primary outcome is the DASH score at 1 year while under observation, regardless of deviations from assigned treatment or other concomitant treatments. The noninferiority limit will be tested at a 1-sided alpha = 0.025.

The primary analysis will use linear regression for all observed post-randomization measurements, with the participant as a random effect, and the baseline DASH score, randomized treatment, visit number, and the treatment–visit interaction as fixed covariates. The results are presented as the estimated mean difference in the DASH score at 1 year with 95% confidence intervals, the 1-sided *p* value for noninferiority, and the 2-sided *p* value for differences from zero.

The primary model is unbiased for data that are missing at random conditional on the treatment arm, baseline, and a normally distributed individual baseline- and visit-adjusted mean DASH. The impact of missing data will be investigated via sensitivity analyses, which assume a continuum of nonrandom differences between observed and missing data. No imputation will be used.

Supplementary analyses will target the biological efficacy of nonsurgical versus surgical treatment through adjusted per-protocol analyses.

Secondary outcomes will be interpreted as supportive of the primary and analyzed without formal multiplicity adjustment. DASH comparisons for nonprimary timepoints will be taken from the primary analysis. VAS data will be analyzed via the same method as the primary analysis (adjusting for their respective baseline). Likert-scale outcomes will be analyzed via proportional odds logit regression adjusted for baseline as a categorical variable, separately for each timepoint. Complications, radiographic healing, and return to work and activities will be presented descriptively. A detailed statistical analysis plan (SAP) will be finalized before analysis.

### Blinding

As this is a surgical vs nonsurgical treatment trial, it is not feasible to blind patients or surgeons to the treatment allocation. The statistical analysis will be performed by a blinded biostatistician not connected to the project apart from the statistical analysis.

### Interim analysis

A blinded sample size re-estimation [63] will be performed in the case of slow recruitment to determine if the target sample size can be adjusted. The inclusion rate will be monitored by the Data and Safety Monitoring Committee (DSMC) (composition is outlined below) as well as the TSC.

There will not be an interim analysis on the primary outcome (DASH), and the reasoning is that no study has as of yet indicated a clinically significant difference in outcome between surgical and nonsurgical treatment. However, the DSMC will monitor the complication frequency in both treatment arms at predefined intervals and may advise the TSC on premature termination of the study if there is a gross imbalance in complication frequency. The DSMC will schedule a meeting shortly after study enrollment begins to agree on monitoring frequency. The DSMC will operate independently from the rest of the study group.

## Protocol violation

Divergence from the protocol reduces the quality or completeness of the data, impacts the safety or health of the participants, or invalidates the informed consent form. Examples include the following: lack of informed consent; inappropriate inclusion or exclusion; unreported AEs or SAEs; inadequate record keeping, including the handling of identification data; noncompliance from the participants; and intentional deviations from the protocol, regulations, or good clinical practice on the part of the study personnel.

Protocol violation also occurs if any of the following occurs:

- Loss to follow-up.
- Treatment crossover outside the predefined period.

Patients who meet these criteria will remain in the study but will be omitted from the per-protocol analysis.

## Participant withdrawal

If a patient withdraws their consent, they can still be included in the statistical analysis if baseline data have been collected, and the impact of missing data will be analyzed in sensitivity analyses as described in the Statistical analysis section above.

## Data management and safety

The PROM questionnaires will be sent out by e-mail, but physical copies will be available. A researcher will check for completeness in REDCap and review them for missing data. If data are missing, the worker, without interference from the medical staff, will assist the participants in completing the questionnaires. Clinician-assessed data are fed directly into REDCap by a physician active in the study at each follow-up visit. Study-related information is stored in REDCap, which is protected with secure firewalls and requires authentication for access. Access will be restricted to trial workers currently working on the trial, and their access will be restricted to data entry. The principal investigator (PI) will keep a list of all persons with access. Data will be pseudonymized prior to analysis and publication.

## Discussion

The ScanDiLaC study will compare the effectiveness of surgical versus nonsurgical treatment of displaced Neer type II and V lateral clavicle fractures and attempt to define the appropriate timing of early crossover surgery. The optimal treatment for the unstable Neer types II and V is not clear from the literature.

The TSC made a mutual, pragmatic decision to not include patients above 65 years old in the study, as no one would consider performing primary surgical treatment for this age group.

To achieve sufficient power in this study, we would need to ensure an adequate inclusion rate. In 2024, 320 displaced LCFs were registered in the Swedish Fracture Register [64] across all of Sweden. If only the currently recruiting sites are included and consider the possibility of incorrectly registered fractures [65], as well as the expected success rate of inclusion for each patient, we have estimated an optimistic possible inclusion rate of 31–32 patients per year in Sweden. We expect that the number will be matched when Denmark, Norway, and Finland are combined. This emphasizes the need for this multicenter collaboration between Nordic countries, where there are comparable healthcare systems and cultural and demographic similarities. Despite this, there are local differences in treatment, particularly with surgical treatment, where the use of hook plates has been a topic of much debate among orthopedic surgeons [18–21]. Many sites use hook plates, whereas others do not. This leads us to take a more pragmatic approach and allow both hook plates and anatomical locking plates with or without CC fixation. Since most surgeons consider removal of the hook plate after bony healing to be a mandatory part of the treatment algorithm [19], this will not be considered a complication in the context of this study.

Given the possibility of surgical treatment differing between sites, there is a risk of bias in the surgical group where it would be possible that a single site only randomizes to surgical treatment. To minimize this risk, the randomization will be stratified by sites in smaller permuted blocks of 2 and 4 to limit the risk of open blocks in multiple sites, as the expected number of patients included per site is low. This reduces the risk of allocation bias.

The early identification of nonunion, symptomatic delayed union, and subsequent treatment is not properly studied in the context of LCFs. There is evidence that for midshaft clavicle fractures, a minimal decrease in pain between 2 and 4 weeks after nonsurgical treatment is indicative of a high risk of symptomatic nonunion [34]. It is the experience and belief of the TSC that this can be extrapolated to encompass LCFs as well and apply severe persisting pain and the absence of radiographic healing at the 6-week follow-up as a criterion for offering the participant crossover to surgical treatment.

The decision to use the DASH score as our primary outcome is because it is one of the most widely used and validated questionnaires for symptoms and function in the upper extremity [36–38]. There are several problems with DASH; however, one is that it does not safely differentiate between the injured and uninjured arms and has been shown to be slightly influenced by disorders in the lower limb as well [66]. Because of this, the TSC decided to add same-time upper extremity injury estimated to affect the primary outcome as an exclusion criterion. Since the DASH may be considered a general PROM, we decided to use the clavicle-specific NCS, translated into Swedish, Danish, Norwegian, and Finnish versions, as a disease-specific secondary outcome, as recommended by Padua et al [67]. The NCS is, at the time of writing, currently being validated in Swedish but will not be validated in other languages.

The ScanDiLaC trial is a multicenter RCT that may provide a better understanding of the differences in outcomes of nonsurgical and surgical treatment of displaced Neer type II and V lateral clavicle fractures while reflecting real-world clinical practice and guiding the development of a treatment algorithm for the orthopedic community.

## Trial status

Protocol version 1.3, updated 9 December 2025. Recruitment began 1 September 2025. Recruitment completion estimated to 31 December 2028. Protocol amendments are either internally discussed among the TSC or submitted for review by the relevant Research Ethics Committees, depending on the impact of the amendment.

## Supplementary Information

Supplementary Material 1.

Supplementary Material 2.

Supplementary Material 3.

Supplementary Material 4.

Supplementary Material 5.

## Data Availability

Uppsala University is the sponsor of the trial and is responsible for data protection and management. Study-related information is secured in REDCap, which requires two-factor authentication and data logging for managing databases. Access will be limited to trial workers currently working on the trial, whose access will be limited to data entry only. A list of persons with access will be kept by the PI. No identifiable personal data will be published. Trial results are planned to be published in an international peer-reviewed journal.

## Abbreviations

ScanDiLaC: Scandinavian Displaced Lateral Clavicle trial
RCT: Randomized controlled trial
DASH: Disabilities of the Arm, Shoulder and Hand
EQ-5D-5L: European Quality of Life, 5 Dimensions, 5 Levels
UCLA: University of California, Los Angeles
NCS: Nottingham Clavicle Score
VAS: Visual analog scale
PGIC: Patient Global Impression of Change
LCF: Lateral clavicle fracture
CC: Coracoclavicular
PROM: Patient-reported outcome measure
SPIRIT: Standard Protocol Items: Recommendations for Interventional Trials
TSC: Trial Steering Committee
REDCap: Research Electronic Data Capture
TIDieR: Template for Intervention, Description and Replication
ASA: American Society of Anesthesiologists
AE: Adverse event
SAE: Serious adverse event
CUA: Cost-utility analysis
QALY: Quality-adjusted life years
NICE: National Institute for Health and Clinical Excellence
SD: Standard deviation
MCID: Minimal clinically important difference
SAP: Statistical analysis plan
DSMC: Data and Safety Monitoring Committee
PI: Principal investigator
ICMJE: International Committee of Medical Journals Editors
