# Supplementary figures and images for: The Scandinavian Displaced Lateral Clavicle trial (ScanDiLaC): a study protocol for a randomized clinical trial
Source: Trials. 2026 Jun 13;27:438. doi: 10.1186/s13063-026-09844-8 (PMC13263930; doi:10.1186/s13063-026-09844-8)

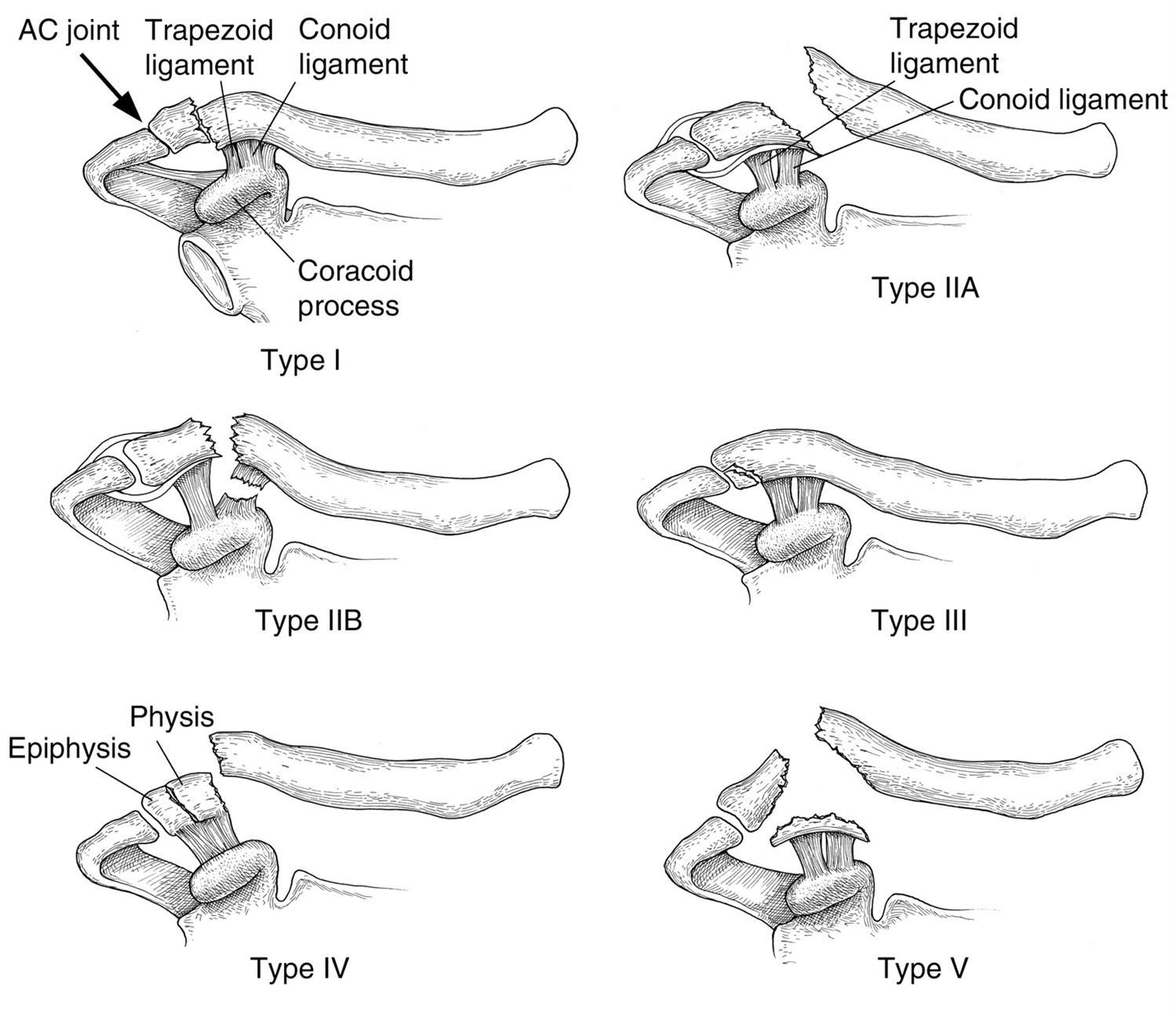

Supplement: Supplementary file 1 — Supplementary Material 1. [file 13063_2026_9844_MOESM1_ESM.jpg]
