# Supplementary material for: The Scandinavian Displaced Lateral Clavicle trial (ScanDiLaC): a study protocol for a randomized clinical trial
Source: Trials. 2026 Jun 13;27:438. doi: 10.1186/s13063-026-09844-8 (PMC13263930; doi:10.1186/s13063-026-09844-8)
Supplement: Supplementary file 2 — Supplementary Material 2. [file 13063_2026_9844_MOESM2_ESM.pdf]

Sweden	Denmark	Finland	Norway
Uppsala University Hospital	Copenhagen University Hospital Hvidovre	Tampere University Hospital	Østfold Hospital Trust
Umeå University Hospital	Odense University Hospital		
Linköping University Hospital	Zealand University Hospital		
Karolinska University Hospital	National Hospital of the Faroe Islands		
Stockholm South Hospital			
Danderyd Hospital			
Örebro University Hospital			
Malmö University Hospital			
