# Supplementary material for: The Scandinavian Displaced Lateral Clavicle trial (ScanDiLaC): a study protocol for a randomized clinical trial
Source: Trials. 2026 Jun 13;27:438. doi: 10.1186/s13063-026-09844-8 (PMC13263930; doi:10.1186/s13063-026-09844-8)
Supplement: Supplementary file 3 — Supplementary Material 3. [file 13063_2026_9844_MOESM3_ESM.pdf]

The New Berlin definition of polytrauma

For the purpose of the ScanDiLaC study, polytrauma will be defined accordin to the New Berlin definition as:

1. Abbreviated Injury Score (AIS) of ≥ 3 in **two or more** of the six body regions (head/neck, face, thorax, abdomen, limb and external) – see table below

**AIS**	Severity
**1**	Minor
**2**	Moderate
**3**	Serious, not life threatening
**4**	Severe, life threatening
**5**	Critical, survival uncertain
**6**	Virtually unsurvivable

2. **One or more** additional variables from five physiologic parameters:
   - **a.** Hypotension (≤ 90 mmHg systolic blood pressure)
   - **b.** Unconsciousness (Glasgow Coma Scale ≤ 8)
   - **c.** Acidosis (base excess ≤ -6.0)
   - **d.** Coagulopathy (international normalized ratio (INR) ≥ 1.4)
   - **e.** Age (≥ 70 years)
