# Supplementary material for: The Scandinavian Displaced Lateral Clavicle trial (ScanDiLaC): a study protocol for a randomized clinical trial
Source: Trials. 2026 Jun 13;27:438. doi: 10.1186/s13063-026-09844-8 (PMC13263930; doi:10.1186/s13063-026-09844-8)
Supplement: Supplementary file 4 — Supplementary Material 4. [file 13063_2026_9844_MOESM4_ESM.pdf]

Physiotherapy program

ScanDiLaC-study

Based on the Swedish Axelina© rehab program

Initial phase (week 0-3)

Goals	Optimize healing, avoid stiffness.<br>Pain relief
Restrictions	No painful activities<br>No heavy loads
Sling/collar'n'cuff	Used for pain relief 1-2 weeks<br>May be removed for physical therapy or hygiene
Physical therapy	- Posture training, finding the correct scapular position<br>- Taping the scapula in the correct position if difficult<br>- Actively unloaded range of motion exercises in the scapular plane (30 degrees from the frontal plane) as pain allows<br>- Core- and scapular control<br>- Choose exercises from the LIGHT ("LÄTTA") category for range of motion ("rörlighet") and scapula ("skapula")
To next phase	When pain-free in range of motion below shoulder level

Build-up phase (week 4-8)

Goals	Increasing range of motion with good muscle control
Restrictions	No painful activities<br>No heavy loads
Physical therapy	- Active range of motion exercises with good core- and scapular control, focusing on scapular stability<br>- Light dynamic training of scapular muscles<br>- Light dynamic rotator cuff training<br>- Choose exercises from the MEDIUM ("MEDEL") category for range of motion and LIGHT/MEDIUM ("LÄTTA/MEDEL") for scapula and rotator cuff
To next phase	When pain-free in daily activity

Return phase (week 9-12)

Goals	Normalized function
Restrictions	No training or activity causing pain
Physical therapy	- Increased/continued scapular stability<br>- Successively increased loads<br>- Stretching as needed<br>- Choose exercises from the MEDIUM/ADVANCED ("MEDEL"/"AVANCERAD") category for scapula and rotator cuff
