# Supplementary material for: The Scandinavian Displaced Lateral Clavicle trial (ScanDiLaC): a study protocol for a randomized clinical trial
Source: Trials. 2026 Jun 13;27:438. doi: 10.1186/s13063-026-09844-8 (PMC13263930; doi:10.1186/s13063-026-09844-8)

AXELINAS HEMÖVNINGAR ENLIGT SVÅRIGHETSGRAD

RÖRLIGHET — LÄTT

Flexion/elevation

P1

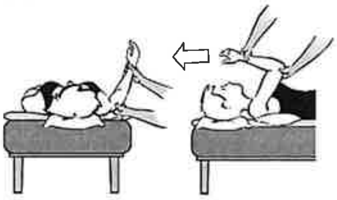

P3

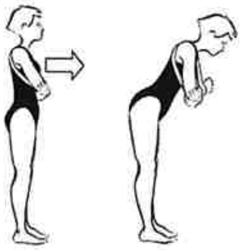

P3X

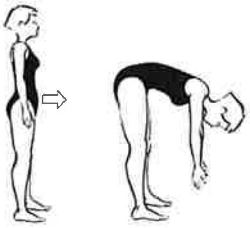

AA19

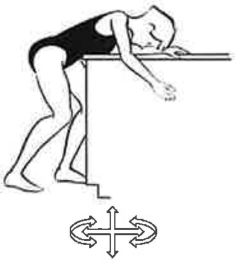

AA12

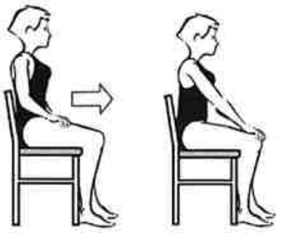

AA12X

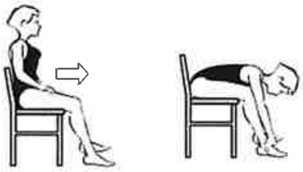

RÖRLIGHET — MEDEL

Flexion/elevation

AA13

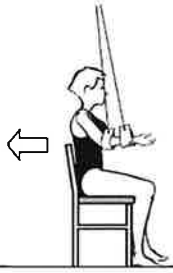

AA15

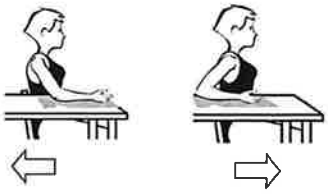

AA17

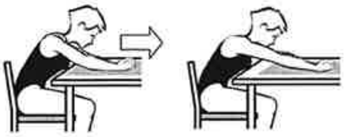

AA1

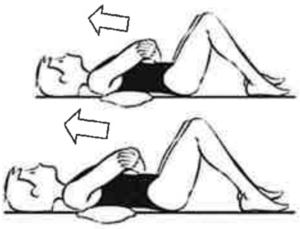

AA2

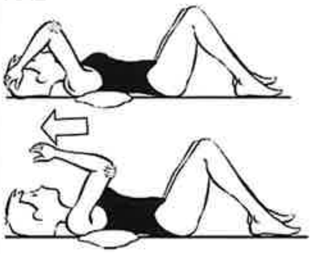

AA3

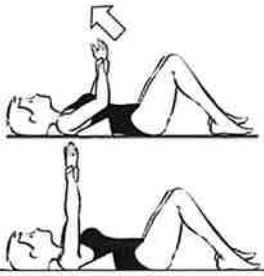

AA6

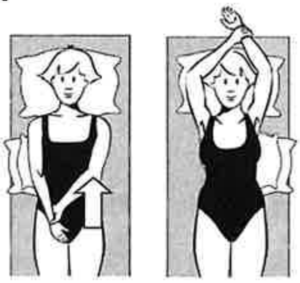

AA4

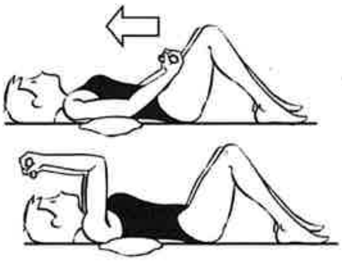

AA5

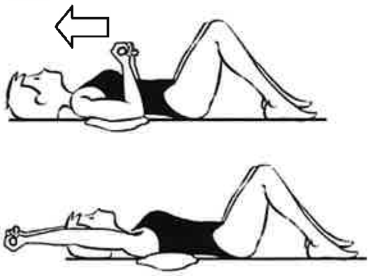

AXELINAS HEMÖVNINGAR ENLIGT SVÅRIGHETSGRAD

RÖRLIGHET — MEDEL, *forts*

Flexion/elevation

AA18

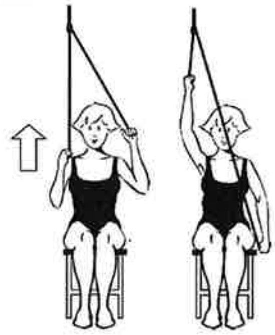

AA20

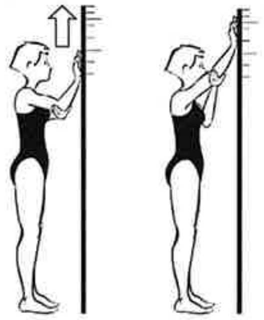

A5

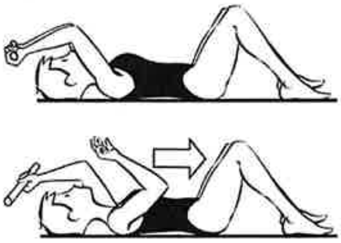

A7

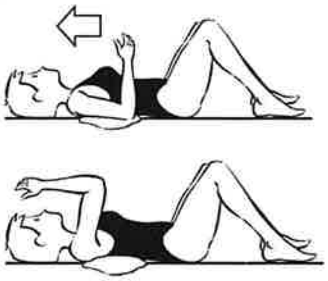

A22

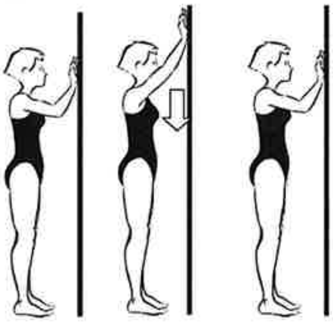

RÖRLIGHET — AVANCERAD

Flexion/elevation

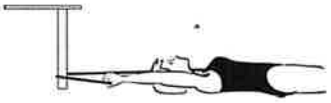

S2

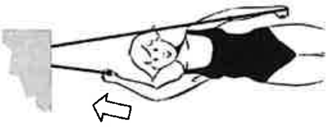

S10

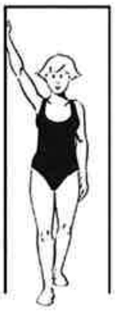

S11

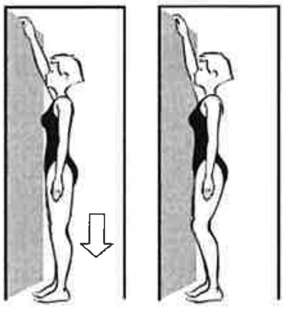

A18

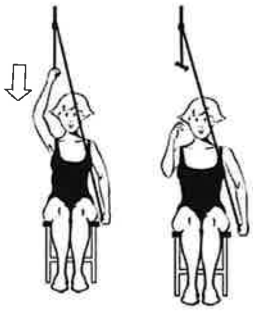

AXELINAS HEMÖVNINGAR ENLIGT SVÅRIGHETSGRAD

RÖRLIGHET — LÄTT

Abduktion

AA7

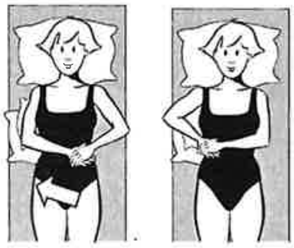

AA8

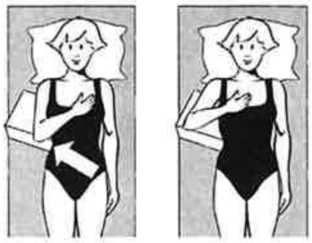

AA14

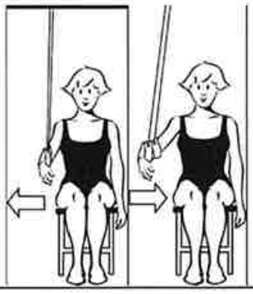

AA16

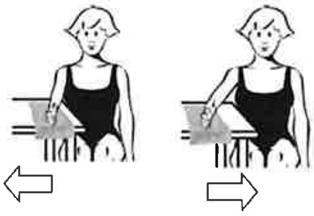

RÖRLIGHET — MEDEL

Abduktion

S1

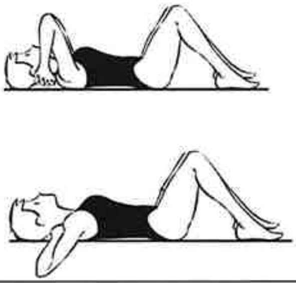

AA21

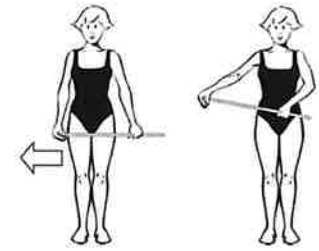

A17

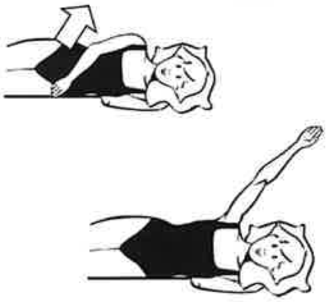

A24

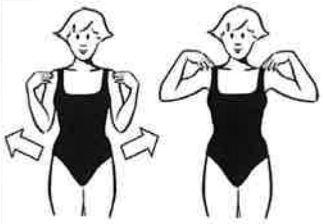

A22

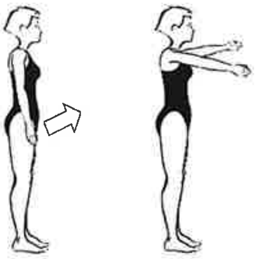

RÖRLIGHET — AVANCERAD

Abduktion

S2

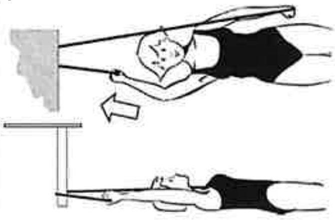

S5

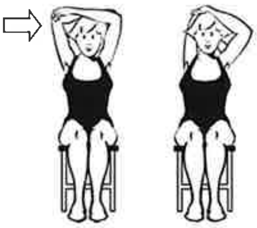

AXELINAS HEMÖVNINGAR ENLIGT SVÅRIGHETSGRAD

RÖRLIGHET — LÄTT

Utåtrotation

P2

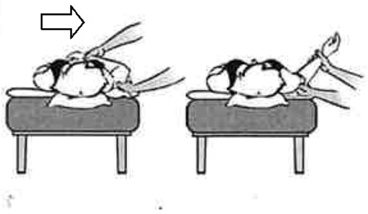

AA9

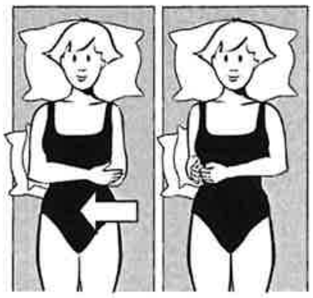

AA10

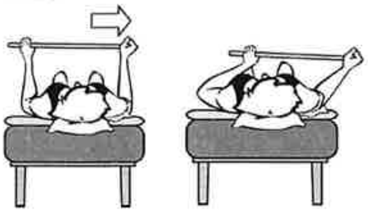

RÖRLIGHET — MEDEL

Utåtrotation

AA11

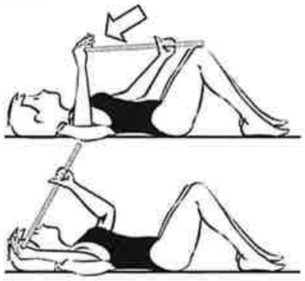

AA22

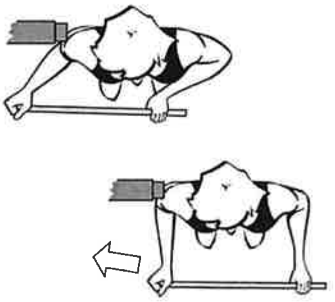

AA22X

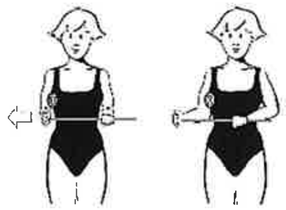

A4

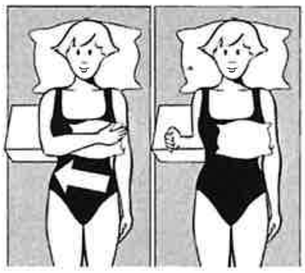

AA22XX

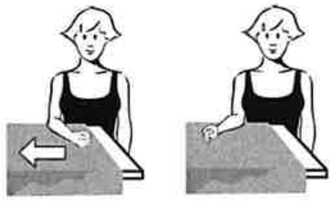

A27

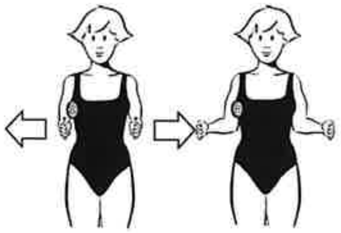

RÖRLIGHET — AVANCERAD

Utåtrotation

S8

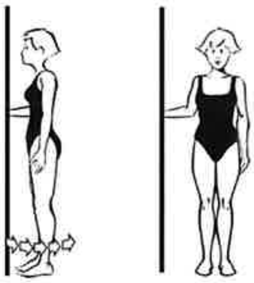

AXELINAS HEMÖVNINGAR ENLIGT SVÅRIGHETSGRAD

RÖRLIGHET	LÄTT	
**Inåtrotation**		
C10 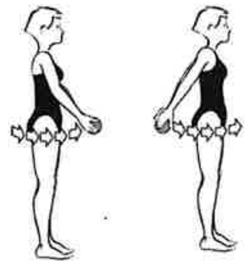	AA24 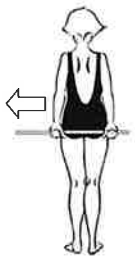	AA25 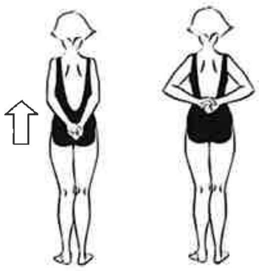

RÖRLIGHET	MEDEL	
**Inåtrotation**		
AA26 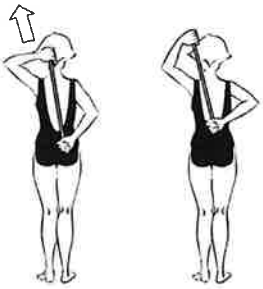	S12 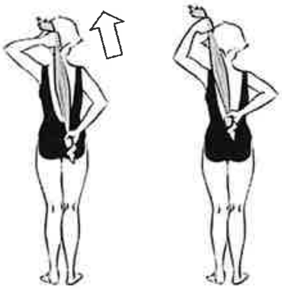	

RÖRLIGHET	AVANCERAD	
**Inåtrotation**		
S12X 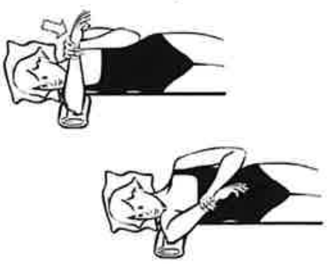		

RÖRLIGHET	AVANCERAD	
**Adduktion**		
S6 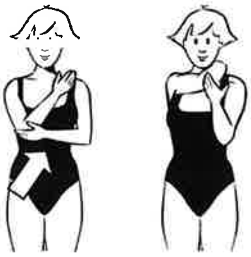	S7 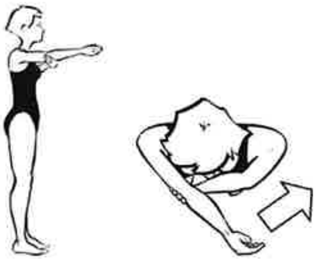	

AXELINAS HEMÖVNINGAR ENLIGT SVÅRIGHETSGRAD

SKAPULA	LÄTT	
BP9 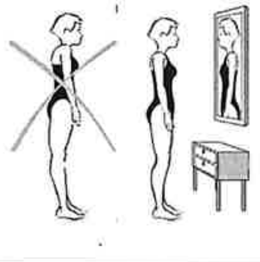	A19 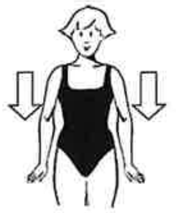	A20 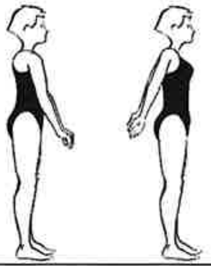
A20X 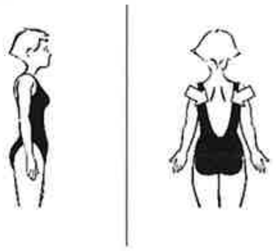	BP7 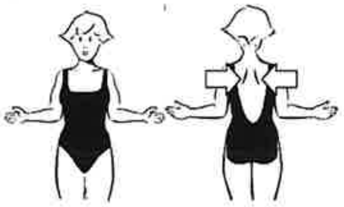	BP7X 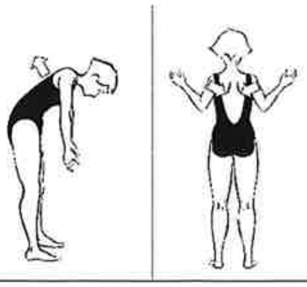
I3 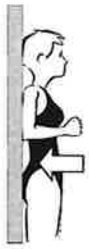	I3X 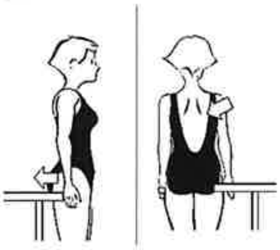	A6 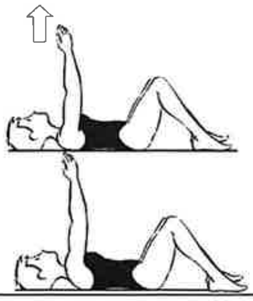
A10 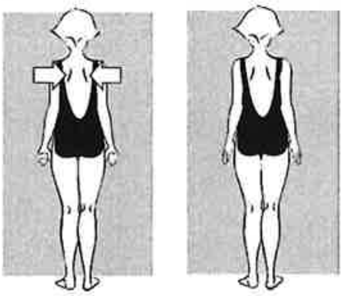	A16 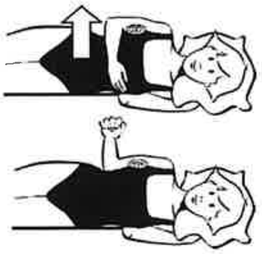	A12 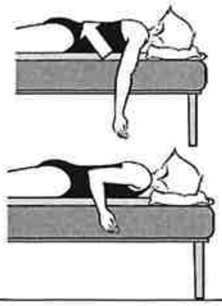
A11 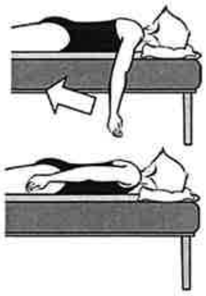	A17 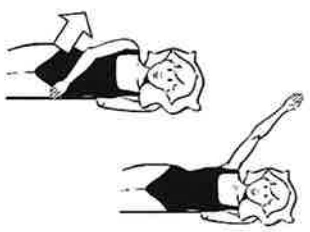	A23 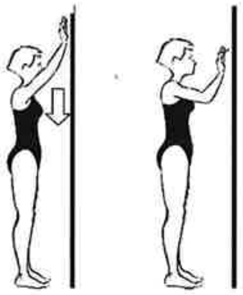

AXELINAS HEMÖVNINGAR ENLIGT SVÅRIGHETSGRAD

SKAPULA	LÄTT, *forts*	
A23X 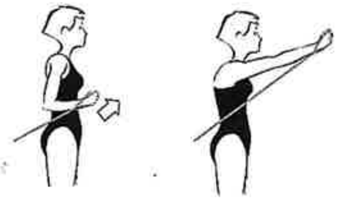	A23XX 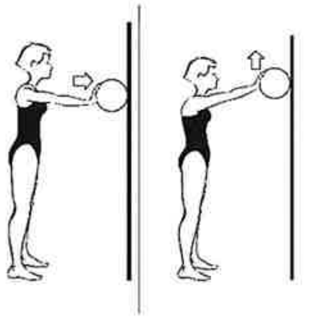	C19 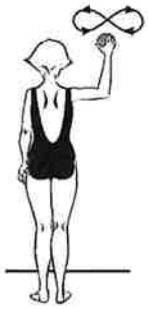
RB1 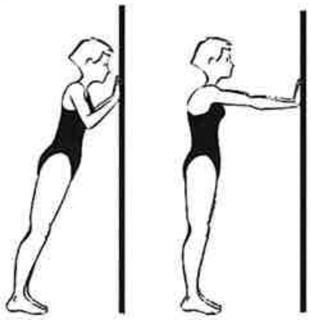		

AXELINAS HEMÖVNINGAR ENLIGT SVÅRIGHETSGRAD

SKAPULA MEDEL

A13	A14	A15
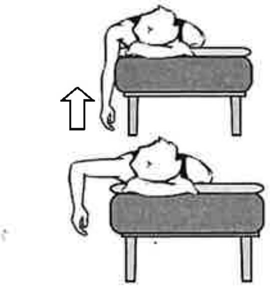	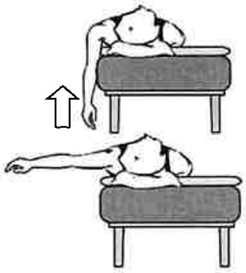	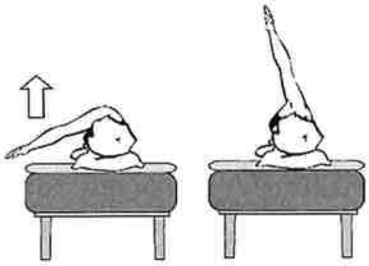
**RE14**	**RE15**	**RE22**
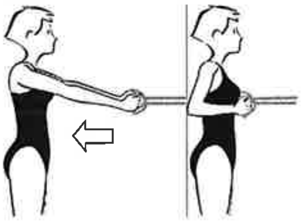	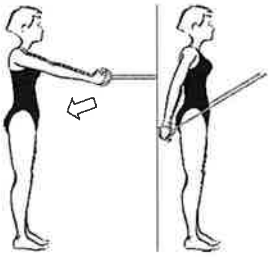	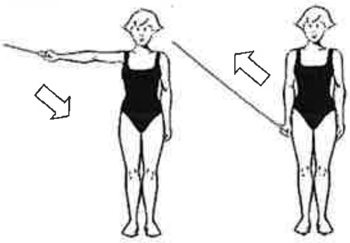
**RE27**	**RW8**	**RW10**
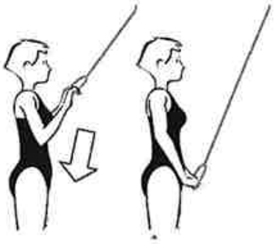	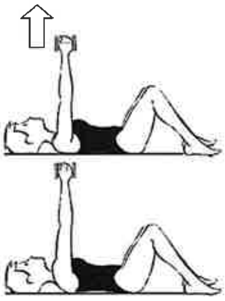	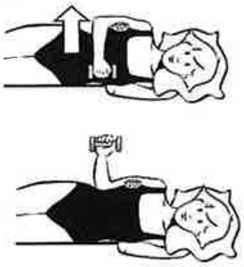
**RB2**	**RE16**	**RE17**
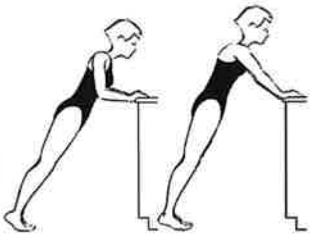	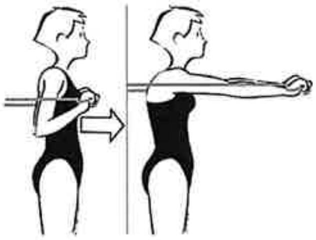	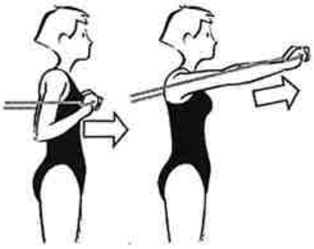
**RW2**	**RW3**	**RW4**
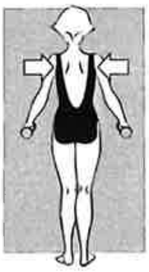	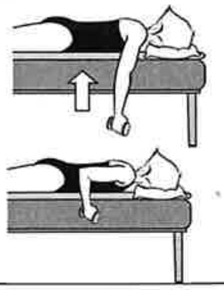	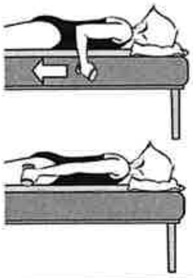

AXELINAS HEMÖVNINGAR ENLIGT SVÅRIGHETSGRAD

SKAPULA	MEDEL, *forts*	
RW1 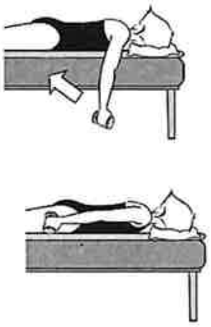	A9 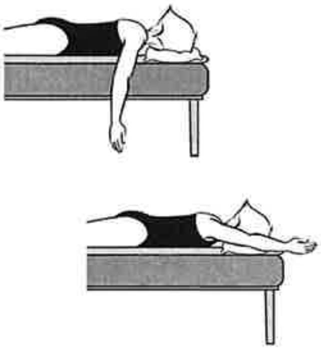	RE26 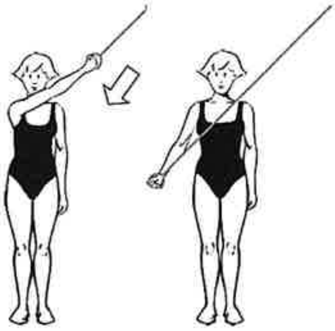

AXELINAS HEMÖVNINGAR ENLIGT SVÅRIGHETSGRAD

SKAPULA — AVANCERAT

RE23 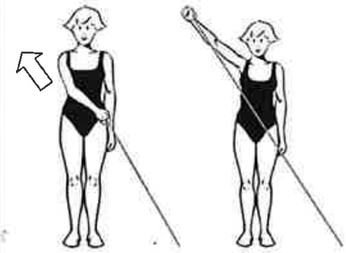	RE24 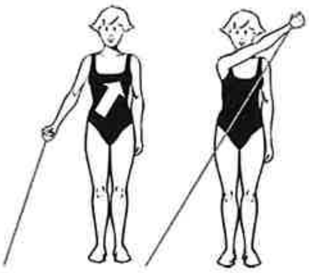	RE25 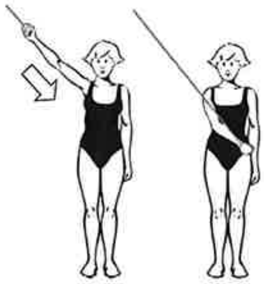
RB3 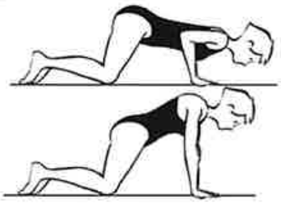	C2 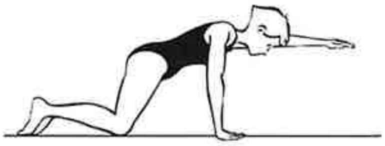	C3 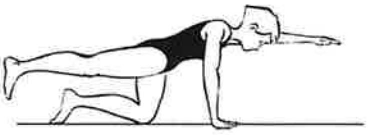
RW5 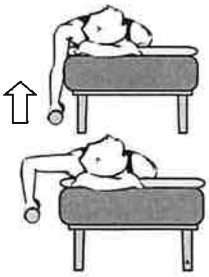	RW6 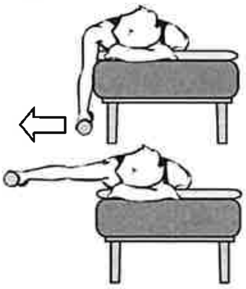	RW11 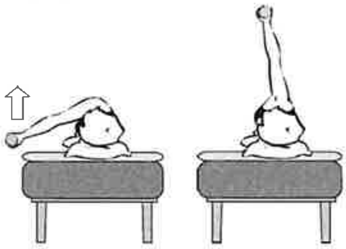
RB1X 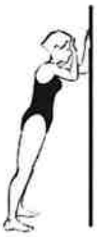	RE16X 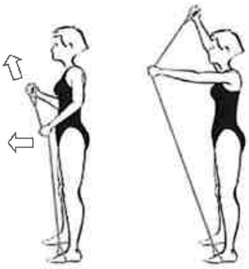	RW7 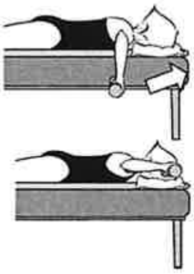
RW7X 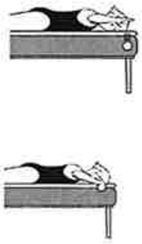	C16 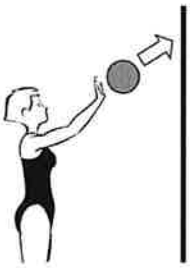	C17 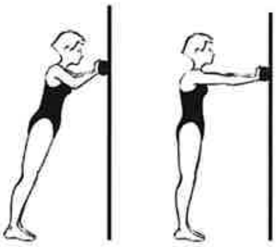

AXELINAS HEMÖVNINGAR ENLIGT SVÅRIGHETSGRAD

SKAPULA	AVANCERAT, *forts*	
RB5 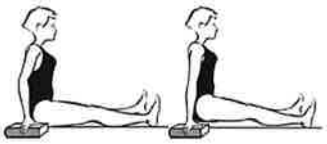	C4 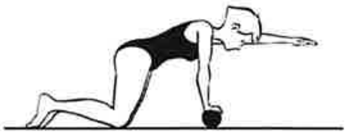	C5 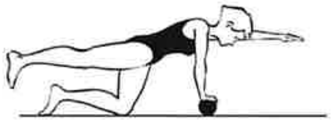
RB4 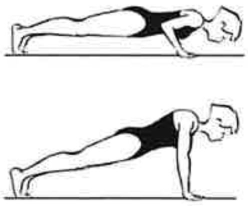	RB4X 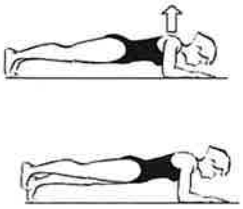	

AXELINAS HEMÖVNINGAR ENLIGT SVÅRIGHETSGRAD

ROTATORCUFF — LÄTT

Abduktion

AA8	AA14	AA16
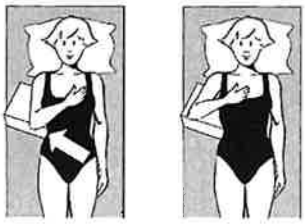	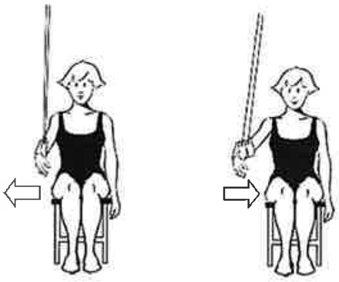	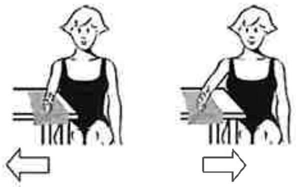
I4	I5	
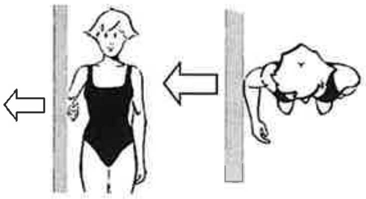	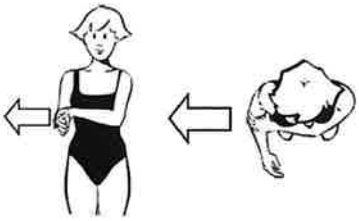	

ROTATORCUFF — MEDEL

Abduktion

A17X	A17XX	RE13
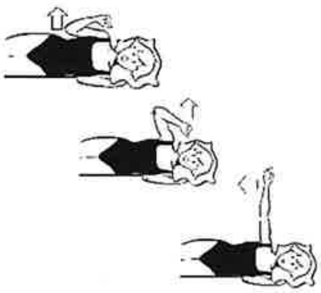	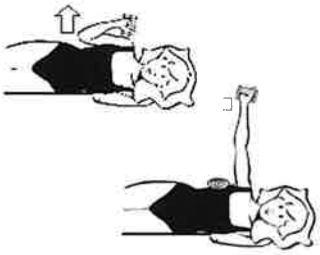	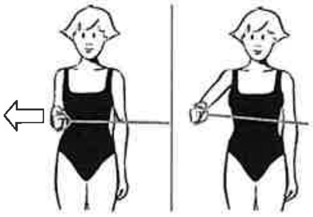

AXELINAS HEMÖVNINGAR ENLIGT SVÅRIGHETSGRAD

ROTATORCUFF — AVANCERAD

Abduktion

RE21 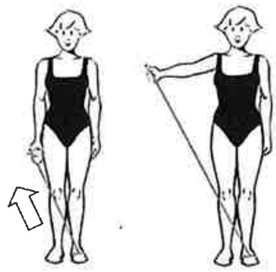	RW13 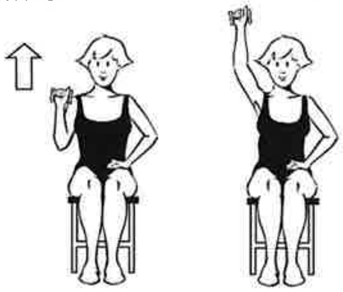	RW15 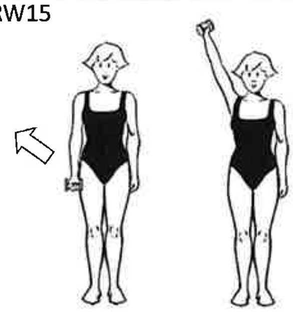
RB8 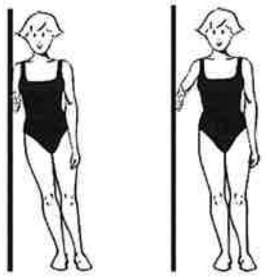	RW15X 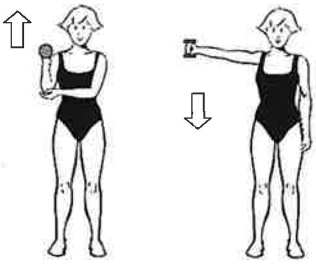	

AXELINAS HEMÖVNINGAR ENLIGT SVÅRIGHETSGRAD

ROTATORCUFF — **LÄTT**

Utåtrotation

AA16XX

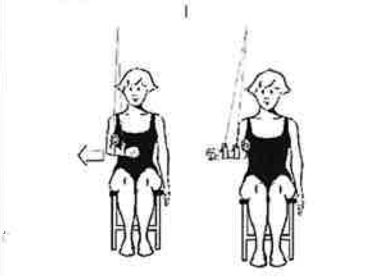

AA16X

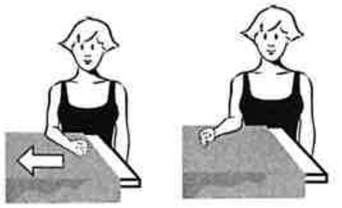

I1

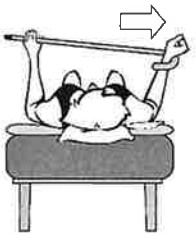

I1X

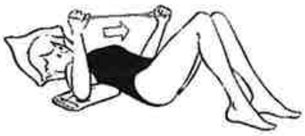

I6

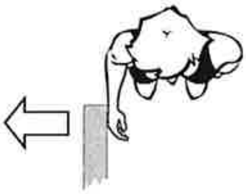

I7

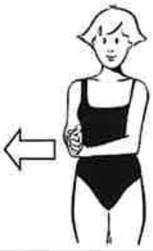

I7X

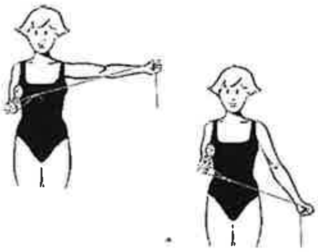

I7XX

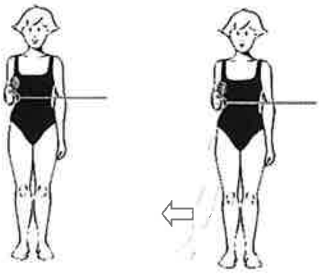

A26

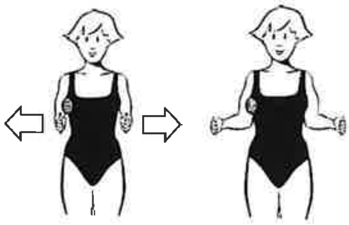

A16

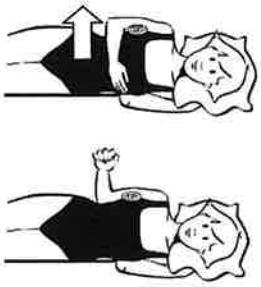

RE9

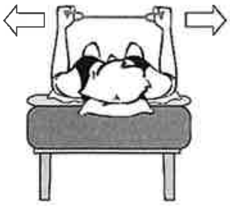

RE9X

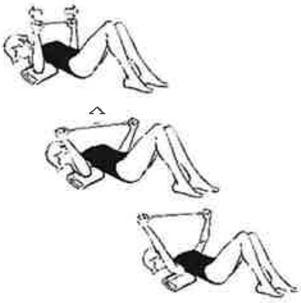

RE9XX

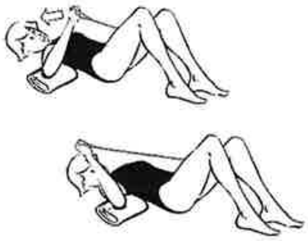

AXELINAS HEMÖVNINGAR ENLIGT SVÅRIGHETSGRAD

ROTATORCUFF — MEDEL

Utåtrotation

RE12X

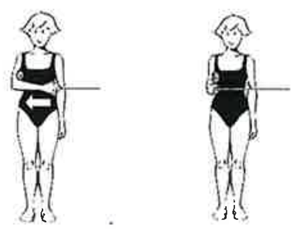

RE12XX

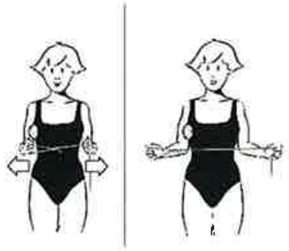

RE12

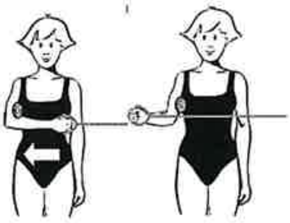

RE6

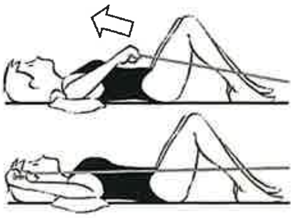

RE8

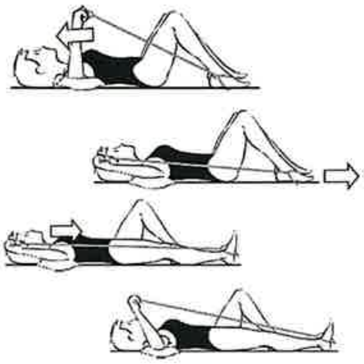

RW9

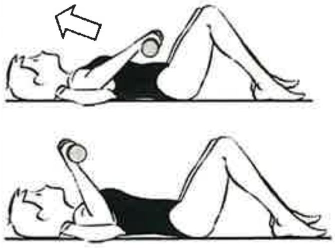

RW10

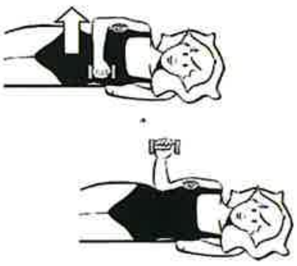

ROTATORCUFF — AVANCERAD

Utåtrotation

RW10X

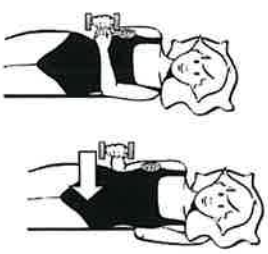

RW12

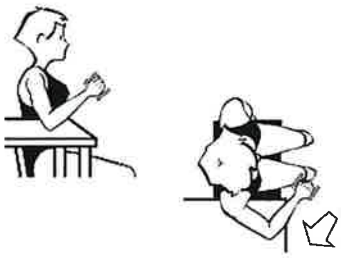

RW12X

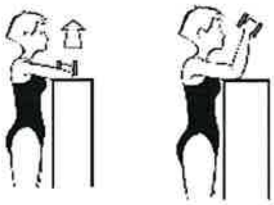

AXELINAS HEMÖVNINGAR ENLIGT SVÅRIGHETSGRAD

ROTATORCUFF — LÄTT

Inåtrotation

I9

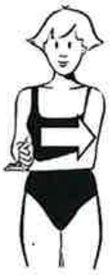

I8

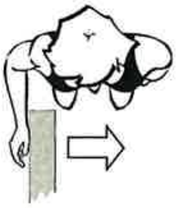

C7

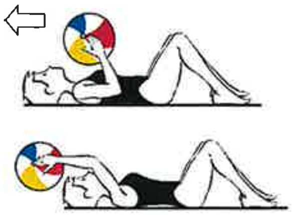

C8

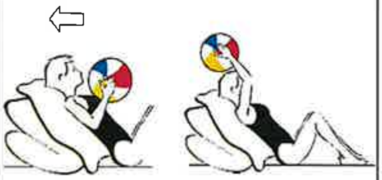

ROTATORCUFF — MEDEL

Inåtrotation

RE7

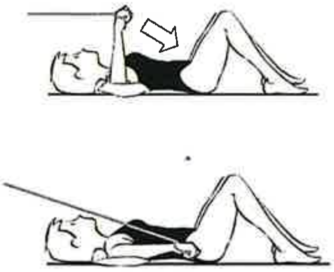

RE11

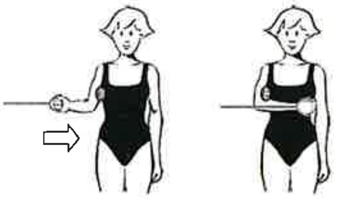

ROTATORCUFF — AVANCERAT

Inåtrotation

RE7

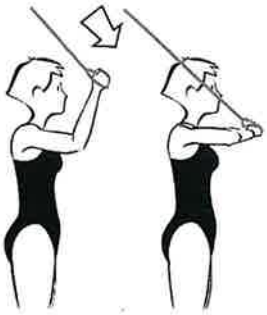

Supplement: Supplementary file 5 — Supplementary Material 5. [file 13063_2026_9844_MOESM5_ESM.pdf]
